# Refined analysis of the *Campylobacter jejuni* iron-dependent/independent Fur- and PerR-transcriptomes

**DOI:** 10.1186/s12864-015-1661-7

**Published:** 2015-07-04

**Authors:** James Butcher, Rebecca A. Handley, Arnoud H. M. van Vliet, Alain Stintzi

**Affiliations:** Department of Biochemistry, Microbiology and Immunology, Ottawa Institute of Systems Biology, University of Ottawa, Ottawa, ON Canada; Institute of Food Research, Gut Health and Food Safety Programme, Norwich Research Park, Norwich, UK

**Keywords:** Fur, PerR, Co-regulation, Iron-independent regulation

## Abstract

**Background:**

The genome of *Campylobacter jejuni* contains two iron activated Fur-family transcriptional regulators, CjFur and CjPerR, which are primarily responsible for regulating iron homeostasis and oxidative stress respectively. Both transcriptional regulators have been previously implicated in regulating diverse functions beyond their primary roles in *C. jejuni*. To further characterize their regulatory networks, RNA-seq was used to define the transcriptional profiles of *C. jejuni* NCTC11168 wild type, Δ*fur*, Δ*perR* and Δ*fur*Δ*perR* isogenic deletion mutants under both iron-replete and iron-limited conditions.

**Results:**

It was found that 202 genes were differentially expressed in at least one mutant under iron-replete conditions and 331 genes were differentially expressed in at least one mutant under iron-limited conditions. The CjFur and CjPerR transcriptomes characterized in this study were compared to those previously identified using microarray profiling and found to be more extensive than previously understood. Interestingly, our results indicate that CjFur/CjPerR appear to co-regulate the expression of flagellar biogenesis genes in an opposing and iron-independent fashion. Moreover the Δ*fur*Δ*perR* isogenic deletion mutant revealed that CjFur and CjPerR can compensate for each other in certain cases, suggesting that both regulators may compete for binding to specific promoters.

**Conclusions:**

The CjFur and CjPerR transcriptomes are larger than previously reported. In particular, deletion of *perR* results in the differential expression of a large group of genes in the absence of iron, suggesting that CjPerR may also regulate genes in an iron-independent manner, similar to what has already been demonstrated with CjFur. Moreover, subsets of genes were found which are only differentially expressed when both CjFur and CjPerR are deleted and includes genes that appear to be simultaneously activated by CjFur and repressed by CjPerR. In particular the iron-independent co-regulation of flagellar biogenesis by CjFur/CjPerR represents a potentially novel regulatory function for these proteins. These findings represent additional modes of co-regulation by these two transcriptional regulators in *C. jejuni*.

**Electronic supplementary material:**

The online version of this article (doi:10.1186/s12864-015-1661-7) contains supplementary material, which is available to authorized users.

## Background

The microaerophilic bacterial pathogen *Campylobacter jejuni* can colonize and thrive in the gut of many different mammalian and avian hosts (humans, bovines, avians, porcines etc.) [1, 2], and *C. jejuni* is also known to persist in the environment during its transit between different hosts [3]. In all these environments, *C. jejuni* must regulate several functions to be able to cope with these variables and often stressful conditions. Previous work has demonstrated that genes relating to iron metabolism and oxidative stress defense are key factors for *C. jejuni*’s survival *in vivo* [2, 4, 5]. The classical iron-regulatory protein in most Gram-negative bacteria, including *C. jejuni*, is the ferric uptake regulator (CjFur) protein, which senses intracellular iron and represses genes involved in iron acquisition accordingly [2, 6–8]. Interestingly *C. jejuni* also possesses a second iron responsive transcriptional regulator, the peroxide responsive regulator PerR that is responsible for regulating *C. jejuni*’s response to peroxide stress [4, 9]. This is in contrast with other gram negative bacteria whose peroxide sensors (e.g. OxyR, OhR) do not utilize iron as a cofactor to sense oxidative stress [10].

It has long been known that the members of the CjFur and CjPerR regulons overlap [9]. The best example of this is the *katA* gene which encodes for catalase. Both CjFur and CjPerR must be deleted for this gene to be fully derepressed and unresponsive to iron repression [9]. In contrast, there are other genes which appear to be solely regulated by either CjFur (e.g. *cfrA*, *chuA*) or CjPerR (e.g. *ahpC*) [2, 4, 8]. At times it has been difficult to clearly define whether a gene is regulated by CjFur and/or CjPerR due to the functional similarity of these two transcriptional regulators. Specifically as Fur family proteins, such as CjPerR, have been shown to use iron or manganese as co-factor *in vitro*, deletion of *fur* may indirectly influence CjPerR activity by altering iron levels in the cell. Moreover, the CjFur and CjPerR binding sequences are likely to be extremely similar based on studies of other organisms harboring multiple Fur family members [11].

There have been several studies that characterized the CjFur and CjPerR transcriptomes using microarray transcriptomic profiling of either Δ*fur* or Δ*perR* isogenic mutant strains [2, 4, 8]. However directly comparing the profiles obtained in these studies can be difficult due to confounding factors that may alter gene expression independently from transcriptional regulator deletion. In *C. jejuni* these can include differences in growth conditions including the gas content used (e.g. gas packs vs. defined gas mixes) and the composition of the growth media [12, 13]. In addition, given that CjFur and CjPerR are known to co-regulate genes, there may be members of the CjFur regulon whose differences in expression are being masked by CjPerR and vice versa. We have therefore characterized the transcriptomes of our *C. jejuni* NCTC11168 wild-type strain and its corresponding Δ*fur*, Δ*perR* and Δ*fur*Δ*perR* isogenic deletion mutants using RNA-seq. These strains were grown following a standardized protocol to improve the reproducibility of the transcriptomic results. While there is increasing evidence that CjFur also regulates genes *in vivo* in the absence of iron (iron-independent regulation) [6], there has been little investigation as to whether CjPerR may also be able to regulate gene transcription in an iron-independent fashion. Thus the transcriptomes of *C. jejuni* NCTC11168 wild-type strain and its corresponding Δ*fur*, Δ*perR* and Δ*fur*Δ*perR* were characterized under iron-replete and iron-limited conditions to define the both iron-dependent /iron-independent transcriptomes of CjFur and CjPerR.

Our results indicate that the CjFur and CjPerR transcriptomes are larger than those previously reported in *C. jejuni* using microarray profiling. This is especially true with regards to iron-independent regulated genes for both CjFur and CjPerR. In addition we have found subsets of genes which are only differentially expressed when both CjFur and CjPerR are deleted and genes which appear to be simultaneously activated by CjFur and repressed by CjPerR.

## Methods

### Bacterial Strains and growth

The *C. jejuni* NCTC11168 wild-type, Δ*fur* [2], Δ*perR* [4] and Δ*fur*Δ*perR* [4] strains were routinely cultured on Mueller-Hinton (MH) agar plates under microaerophilic conditions (83 % N_2_, 4 % H_2_, 8 % O_2_ and 5 % CO_2_) at 37 °C in a MACS-VA500 workstation (Don Whitley, West Yorkshire, England). *C. jejuni* strains were grown overnight in MH agar overlaid with MH broth (biphasic cultures). Prior to inoculation, overnight cultures were washed in minimal essential media alpha (MEMα) (Invitrogen) to remove excess iron sources. Glass flasks used for growing strains for RNA extraction were soaked with 1 M HCl to solubilize bound metal ions and rinsed twice with ddH_2_O prior to sterilization. Strains were grown in a final volume of 50 mL MEMα supplemented with 10 mM pyruvate with freshly prepared FeSO_4_ added at a final concentration of 40 μM as needed to generate iron-replete growth conditions. In agreement with previous work [14], the various strains tested did not show any appreciable differences in their growth under the conditions tested (data not shown). All experiments were done in accordance with the University of Ottawa’s policies for working with biohazardous materials, permit #B05-BMI-1213.

### Total RNA Isolation

*C. jejuni* was grown in MEMα under either iron-replete (+40 μM FeSO_4_) or iron-limited (no added iron) conditions until midlog phase was reached (OD_600_ ~ 0.2). Total RNA was then isolated as described previously [2, 7]. Briefly, a 10 % cold RNA stop solution used to preserve RNA integrity (10 % buffer saturated phenol pH 4.3 in absolute ethanol) was added to the media and cells were pelleted by centrifugation at 6000xg. Pellets were resuspended in TE buffer pH 8.0 and RNA was extracted using a hot-phenol chloroform method [2]. RNA was precipitated overnight at −80 °C and the RNA pellet was washed five times in 80 % ethanol. RNA pellets were resuspended in TE buffer and treated with DNase I (Epicentre) to remove contaminating genomic DNA. Treated samples were purified using the RNeasy kit (Qiagen) and PCR was performed to confirm the absence of genomic DNA. RNA purity and quality was assessed using a Nanodrop and the Experion RNA StdSens analysis kit (Bio-Rad). Samples with high quality RNA were selected for Illumina sequencing library construction. Total RNA was isolated from three independent biological replicates of wild-type, Δ*fur*, Δ*perR* and Δ*fur*Δ*perR* strains under either iron-replete or iron-limited conditions (24 RNA extractions in total).

### Illumina library construction and sequencing

Library construction, cluster generation and sequencing were performed at McGill University and Génome Québec Innovation (Montreal, Canada). Ribosomal RNAs were depleted using the RiboZero Meta-Bacteria kit (Epicentre) and strand-specific sequencing libraries were constructed using TruSeq kits (Epicentre). Cluster generation was done using standard Illumina protocols with sequencing performed on an Illumina HiSeq 2500 sequencer. The libraries were subjected to paired-end sequencing with a target read length of 100 nucleotides and the samples for iron-replete and iron-limited conditions were run on separate lanes (12 samples/lane). At least 12 million reads were obtained per library (Additional file 1: Table S1). The Illumina reads have been submitted to the Sequence Read Archive at NCBI with the accession number SRP044881.

### Read alignment to the *C. jejuni* genome and bioinformatic analysis

**Fig. 1:**
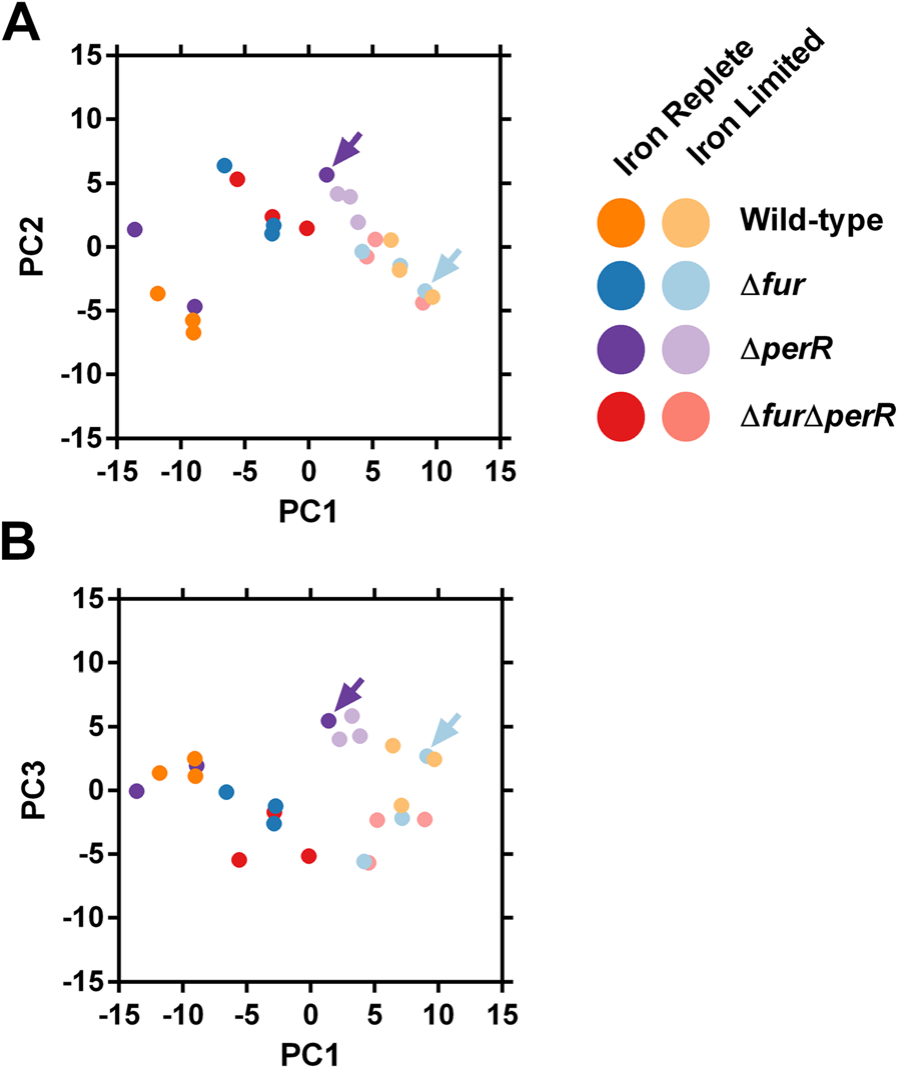
PCA plots of *C. jejuni* transcriptomic samples. The Log(RPKM + 1) values for each gene in the *C. jejuni* NCTC11168 genome were used for PCA analysis and PC1vsPC2 (**a**) and PC1vsPC3 (**b**) were plotted to show the overall structure of the transcriptomic data and to identify sample outliers. Each dot represents a RNA-seq sample with iron-replete samples in dark colors and iron-limited samples in light colors. The PCA showed distinct sample separation based on the iron status of the growth medium highlighting the key influence of iron on the *C. jejuni* transcriptome. The samples for each strain tend to cluster together with two noticeable exceptions in the Δ*perR* iron-replete and Δ*fur* limited groups

**Fig. 2:**
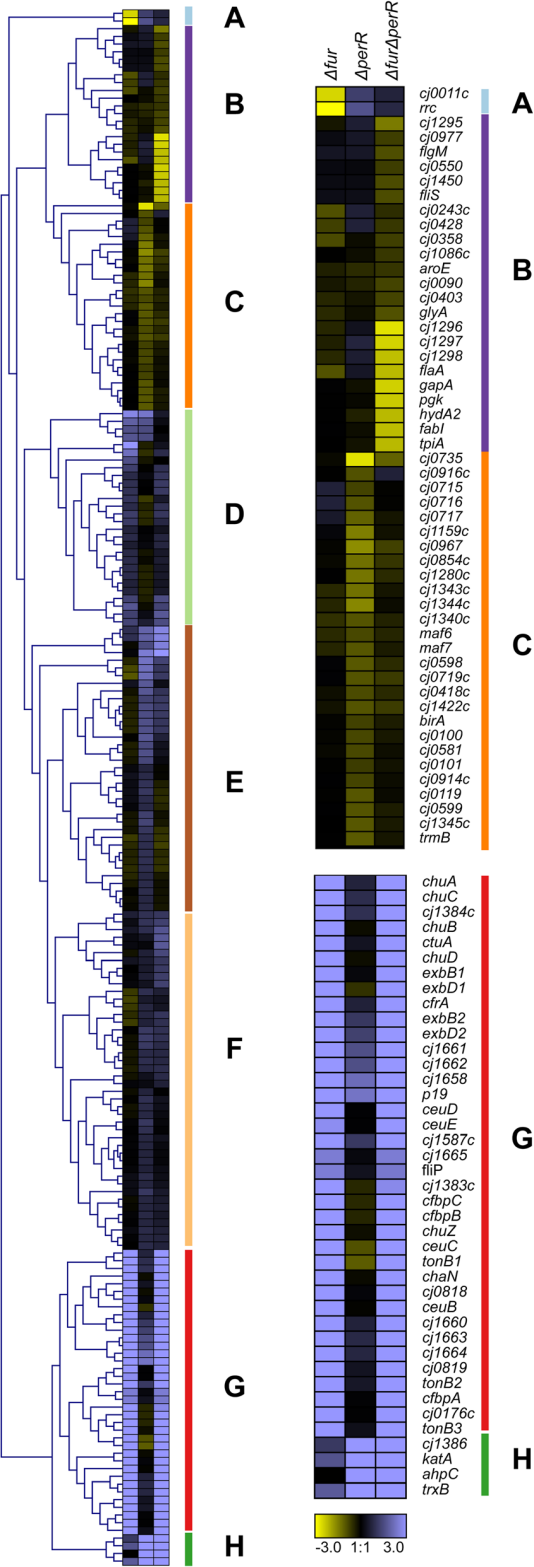
Hierarchical clustering of genes differentially expressed under iron-replete conditions. Genes found to be differentially expressed in at least one strain under iron-replete conditions were subjected to hierarchical clustering in Genesis to identify corresponding genes with similar expression profiles. The columns each represent one strain (Δ*fur*, Δ*perR*, Δ*fur*Δ*perR*) and relative fold changes in expression are presented in a Log_2_ scale with up-regulated genes in blue and down-regulated genes in yellow. The clustering resulted in 8 main clusters (**a**-**h**). Clusters **a**, **b**, **c**, **g** and **h** are expanded to highlight the genes present in each cluster. See Additional file 6: Table S5 for further details and Additional file 8: Figure S2 for full histogram

**Fig. 3:**
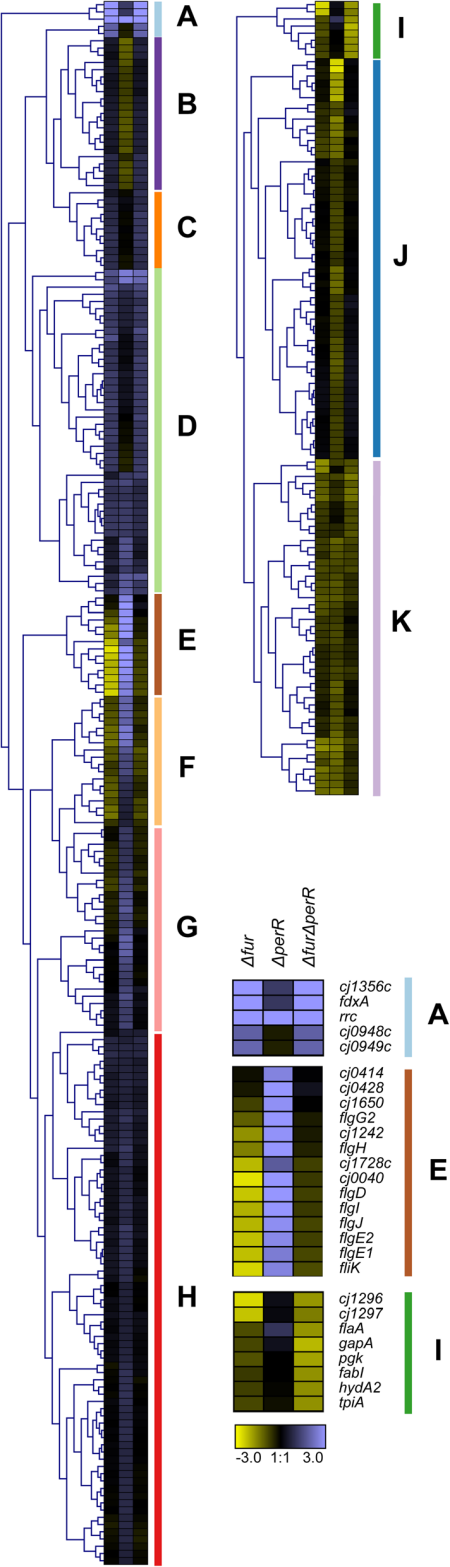
Hierarchical clustering of genes differentially expressed under iron-limited conditions. Genes found to be differentially expressed in at least one strain under iron-limited conditions were subjected to hierarchical clustering to identify corresponding genes with similar expression profiles. Clusters I-K were split from the original clustering figure for ease of viewing. The columns each represent one strain (Δ*fur*, Δ*perR*, Δ*fur*Δ*perR*) and relative fold changes in expression are presented in a Log2 scale with up-regulated genes in blue and down-regulated genes in yellow. The clustering resulted in 11 main clusters (**a**-**k**). Clusters **a**, **e** and **h** are expanded to highlight the genes present in each cluster. See Additional file 7: Table S6 for further details and Additional file 9: Figure S3 for full histogram

Paired end reads were aligned to the *C. jejuni* NCTC11168 genome (NC002163) using Bowtie V2 on the Biolinux platform [15, 16]. Approximately 97 % of reads aligned for each sequencing run (Additional file 1: Table S1). Aligned reads were visualized using Artemis with the Bamview plugin [17]. Raw read counts for each gene were tabulated (Additional file 2: Table S2) using Bedtools and also used to calculate a reads per kilobase coding sequence per million sequenced reads (RPKM) value for each gene (Additional file 3: Table S3). The calculated Log(RPKM + 1) values were used to test the homogeneity of each strain’s biological replicates by performing PCA analysis in R using prcomp [18]. Samples which appeared to cluster differently from their mates were excluded from the downstream differential expression analyses (Fig. 1, Additional file 4: Figure S1). The edgeR statistical package was used to determine gene expression fold changes using the current Genbank gene annotation information for *C. jejuni* NCTC11168 [19, 20]. Transcripts showing a fold change ≥ 1.5 with a FDR value ≥ 0.05 were considered to be significantly differentially expressed (Table 1, Additional file 5: Table S4, Additional file 6: Table S5, Additional file 7: Table S6) [7]. Differentially expressed genes were subsequently merged based on growth condition and clustered (using average linkage Euclidean distance) based on their expression profile using Genesis (Figs. 2 and 3, Additional file 8: Figure S2, Additional file 9: Figure S3) [21]. Differentially expressed genes for each condition were also subjected to gene set enrichment analysis (GSEA) on annotated KEGG pathways using GAGE with a FDR cutoff of <0.1 [22]. Selected significantly enriched pathways were visualized using Pathview [23]. The similarities and differences between the genes found to be differentially expressed in various strains were visualized using hive plots with differentially expressed genes as nodes and genes present in multiple strains connected with ribbons [24]. In addition, genes identified as being part of the Δ*fur* and Δ*perR* transcriptomes via RNA-seq were compared to the Δ*fur* and Δ*perR* transcriptomes previously characterized using microarrays [2, 4]. Genes were considered differentially expressed in the microarrays using the same parameters as in the original papers (>2 fold change, *p* < 0.001). Iron responsive genes were also determined by comparing the wild-type grown under iron-replete and iron-limited conditions (Additional file 10: Table S7) to the iron responsive genes previously identified via microarray profiling or RNA-seq [2, 7].

**Table 1:** Summary of differentially expressed genes in each strain under different growth conditions

	∆*fur*	∆*perR*	∆*fur*∆*perR*
Increased under iron limitation	11	160	49
Decreased under iron limitation	20	113	23
Increased under iron replete conditions	73	85	72
Decreased under iron replete conditions	13	26	23

### Bioinformatic analysis of potential Fur and PerR consensus binding sites

The promoter regions of Fur/PerR regulated genes under either iron-limited or replete conditions were analyzed for the existence of potential binding sites. Genes were analyzed as a whole and also as subgroups based on their potential mode of regulation (iron dependent/independent and activation/repression). Regions consisting of 300 bp upstream the predicted start codon of each gene were retrieved from the RSAT server (http://www.rsat.eu/) [25] and analyzed for potential regulatory motifs by the MEME suite of analysis tools (http://meme-suite.org/) [26]. The genes containing the iron-dependent Fur repression motif consisted of *ceuB*, *cfbpA*, *cfrA*, *chaN*, *chuA*, *chuZ*, *cj0176c*, *cj0818*, *cj1384c*, *cj1587c*, *cj1658*, *dsbI*, *exbB2*, *katA* and *tonB3*.

### RT-qPCR confirmation of selected genes

RT-qPCR was used to confirm the differential expression under iron limited conditions of several genes including *ahpC*, *chuA*, *cj0414*, *cj0415*, *cj1386*, *flgE1*, *flgE2*, *flgG*, *flgH*, and *fliK*. These genes were selected based on the fact that they show inverse regulation by CjFur/CjPerR (*flgE1*, *flgE2*, *flgG*, *flgH*, and *fliK*) or where only differentially expressed in the Δ*perR* mutant (*cj0414*, *cj0415*, *cj1386*). *AhpC* and *chuA* were also included as controls for potential metal dependent regulation by CjPerR or CjFur respectively. RT-qPCR reactions were performed as previously described using *slyD* as an endogenous control [7]. Genes were considered significantly expressed at a fold change >1.5 with a *p* value <0.05.

## Results

### Confirming sample consistency via PCA analysis

Principal component analysis (PCA) was performed to show the overall structure of the transcriptomic data and identify sample outliers based on the growth media (iron-replete vs. deplete) or genetic background (wild-type vs. Δ*fur* vs. Δ*perR* vs. Δ*fur*Δ*perR*). The PCA showed distinct sample separation based on the iron content of the growth medium, highlighting the key influence of iron on the *C. jejuni* transcriptome (Fig. 1a). The Δ*fur*Δ*perR* samples clustered closer to the Δ*fur* samples as compared to the Δ*perR* samples in both iron replete and iron limited conditions. Moreover the samples for each strain tended to cluster together with two noticeable exceptions (Fig. 1a, b, arrows). One of the Δ*perR* samples (perr_plusIron_3) grown in the presence of iron did not group with other iron-replete samples and instead clustered with iron-limited samples (Fig. 1a). In addition one of the Δ*fur* samples (fur_minusIron_2) grown in iron-limited conditions shows a distinctly different clustering pattern from its mates and the Δ*fur*Δ*perR* samples in the PC1vsPC3 plot (Fig. 1b). Therefore both of these outlier samples were removed from downstream differential expression analysis, to avoid introducing artifacts or confounding the identification of differentially expressed genes. To note, including these outlier samples resulted in a significantly reduced number of differentially expressed genes with a high number of false negatives due to increased variation in the gene expression profiles (data not shown).

Previous work in our laboratory has also used RNA-seq to identify iron responsive genes in wild-type *C. jejuni* NCTC11168 [7]. These previous sequencing results were compared to the current wild-type results to determine the similarity of the runs and whether the results from both studies could be merged for meta-analysis. PCA clearly separated the transcriptomes based on the sequencing runs (Additional file 11: Figure S4). In fact, the sequencing results for these two studies could be separated by two axes corresponding to either growth condition (iron-replete vs iron-limited) or study (current vs previous). Therefore to avoid biased results, the iron regulon was reanalyzed using only the data from this study and compared to the iron regulon that was determined previously.

### Transcriptome signatures of the Δ*fur*, Δ*perR* and Δ*fur*Δ*perR* mutants under iron-replete conditions

The major objective of this work was to define the CjFur and CjPerR regulatory networks. Because CjFur and CjPerR regulated genes are known to be iron-repressed we first determined the CjFur and CjPerR regulon under iron-replete conditions as these genes would be differentially expressed between the wild-type and mutant strains. Globally, 86 genes were found to be differentially expressed in the Δ*fur* mutant (73 up-regulated and 13 down-regulated), 111 genes in the Δ*perR* mutant (85 up-regulated and 26 down-regulated) and 95 genes in the Δ*fur*Δ*perR* mutant (72 up-regulated and 23 down-regulated) (Additional file 6: Table S5). Genes found to be differentially expressed in at least one strain were subjected to hierarchical clustering analysis (using average linkage Euclidean distance). The analysis generated 8 main clusters, with clusters A-C representing down-regulated genes and clusters D-H representing up-regulated genes (Additional file 6: Table S5, Fig. 2). Clusters A, B and C contain genes that were primarily down-regulated in the Δ*fur*, Δ*fur*Δ*perR* or Δ*perR* strains respectively. The most down-regulated Δ*fur* genes (Cluster A) consisted of the *rrc*-*cj0011c* operon (29.2 and 5.8 fold activation). Cluster B primarily consists of genes highly down-regulated (~1.5-8 fold) in the Δ*fur*Δ*perR* strain, including *cj1295*-*1298* and several flagellar genes such as *flgM*, *fliS* and *flaA*. Cluster C contains genes primarily down-regulated in the Δ*perR* strain (~2 fold) and includes several motility associated genes (*cj1340c*-*cj1345c*, *maf6*, *maf7*). Interestingly, no genes were found to be down-regulated in all three strains and only *cj1340c* (Δ*fur*, Δ*perR*), *flaA* (Δ*fur*, Δ*fur*Δ*perR*) and *cj1442c* (Δ*perR*, Δ*fur*Δ*perR*) were down-regulated in two of the three strains (Additional file 12: Figure S6).

Clusters D-F consist of genes that were slightly up regulated in at least one of the three strains (~2 fold). In contrast with the down regulated genes, many up regulated genes were up-regulated in multiple strains. Clusters G and h contain genes that were highly up-regulated in at least two strains (~6-120 fold). Genes in cluster g are greatly up-regulated in the Δ*fur* and Δ*fur*Δ*perR* strains. Most of these genes are directly involved in iron acquisition (e.g. hemin: *chuABCDZ*; enterobactin: *cfrA*, *ceuBCDE*; lactoferrin/transferrin: *cfbpABC*, *ctuA*, *chaN*; rhodotorulic acid: *cj1658*-*p19*, *cj1660*-*1665*; ferrous ions: *feoB*,*cj1397*) or supplying the energy required for iron uptake across the outer membrane (e.g. *tonB1*/*B2*/*B3*, *exbB1*/*B2*, *exbD1*/*D2*). Many of these genes have been previously shown to be directly regulated by Fur [8, 27, 28]. Cluster H consists of four genes that are highly up-regulated in all three strains and are involved in oxidative stress defense (*katA*, *cj1386*, *ahpC* and *trxB*). The *C. jejuni* alkyl hydroperoxidase (*ahpC*) has been previously shown to be directly regulated by PerR [29], while the catalase (*katA*), thioredoxin *trxB* and the heme trafficking protein *cj1386* have been suggested to be PerR regulated [4, 9]. In addition *katA*, *trxB* and *cj1386* have been proposed to be Fur regulated [2, 8], although Fur has only been shown to bind the *katA* promoter [7] (note that *katA* and *cj1386* are not co-transcribed [30]). Most of the genes in both clusters G and H have also been previously found to be iron responsive in *C. jejuni* [2, 7, 8].

The promoter regions of differentially expressed genes in each strain (Δ*fur*, Δ*perR* and Δ*fur*Δ*perR*) under each condition (iron-replete and iron-limited) were analyzed by MEME to identify potential binding motifs. However we were only able to identify a consensus binding motif for Fur iron-dependent repressed targets (Additional file 13: Figure S5) that was essentially identical to those already published [2, 6].

### Transcriptome signatures of the Δ*fur*, Δ*perR* and Δ*fur*Δ*perR* mutants under iron-limited conditions

In order to identify genes regulated by CjFur and CjPerR in an iron-independent fashion we also characterized the CjFur and CjPerR transcriptomes under iron-limited conditions. Globally, 31 genes were found to be differentially expressed in the Δ*fur* mutant (11 up-regulated and 20 down-regulated genes), 373 genes in the Δ*perR* mutant (160 up-regulated and 113 down-regulated genes) and 72 genes in the Δ*fur*Δ*perR* mutant (49 up-regulated and 23 down-regulated genes) (Additional file 7: Table S6). Hierarchical clustering analysis of the differentially expressed genes revealed 11 main clusters (A-K) of expression profiles with clusters I-J being primarily composed of genes that were down-regulated in at least one strain and clusters C, D, G and H containing genes that were up-regulated in at least one strain (Fig. 3; ~2-4 fold). Cluster A is composed of genes that were strongly up-regulated in the Δ*fur* and Δ*fur*Δ*perR* strains such as *cj1356c*, *fdxA*, *rrc* and *cj0948c*-*0949c* (~4-8 fold). Cluster I contains genes that were strongly down-regulated in the Δ*fur* and Δ*fur*Δ*perR* strains such as *flaA*, and the flagellar modification island genes *cj1296*-*97* (~3-4 fold).

Cluster B contains genes up-regulated in the Δ*perR* mutant while being down-regulated in the Δ*fur* and Δ*fur*Δ*perR* mutants, while cluster F consists of genes down-regulated in the Δ*perR* mutant while being up-regulated in the Δ*fur* and Δ*fur*Δ*perR* mutants. Interestingly Cluster E is primarily composed of genes that were strongly down-regulated in the Δ*fur* mutant (~3-4 fold down-regulation) and strongly up-regulated in the Δ*perR* mutant (~4-6 fold up-regulation) (also see Additional file 12: Figure S6). These include many different flagellar biogenesis genes such as *flgE1*, *flgE2*, *flgG*, *flgH*, *flgI*, *flgJ*, and *fliK* (to note, most of these genes have been previously shown to be required for *C. jejuni* motility [5, 31]). In addition, none of the genes present in Cluster E were found to be differentially expressed in the Δ*fur*Δ*perR* mutant (Additional file 7: Table S6).

**Fig. 4:**
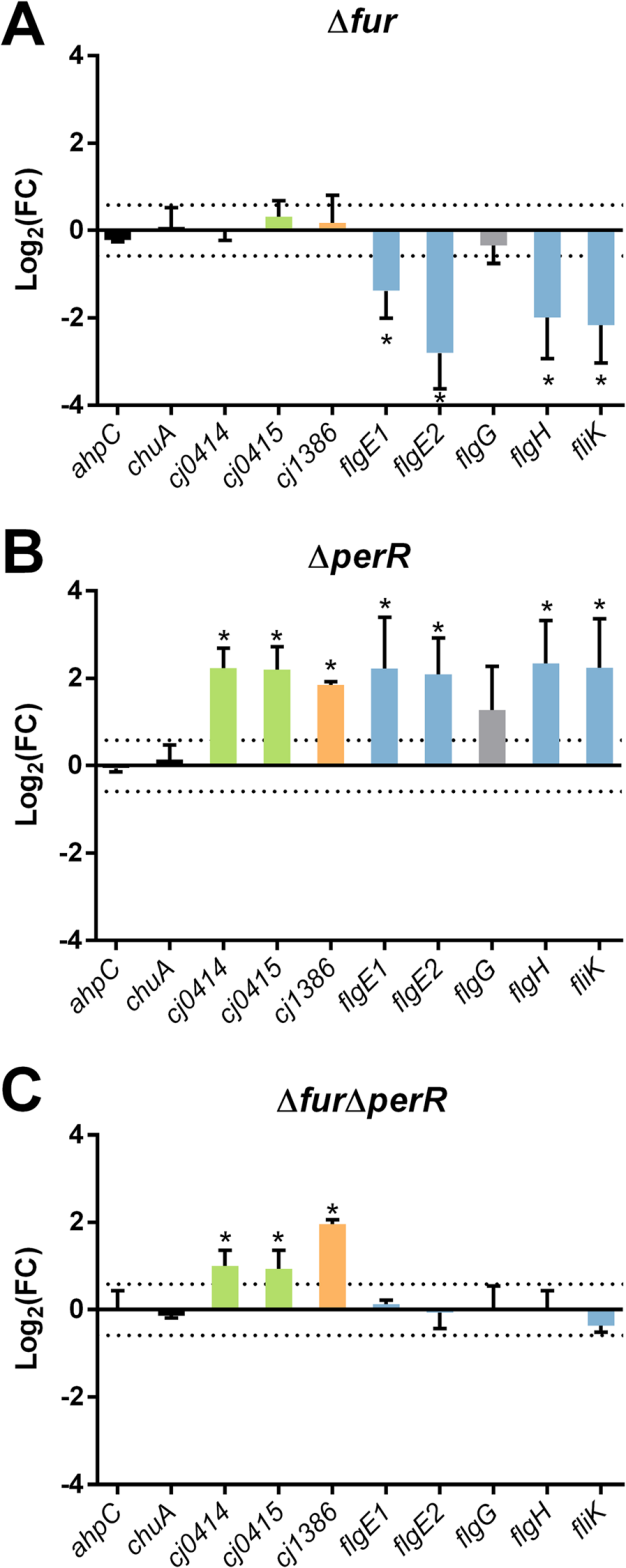
RT-qPCR validation of genes under apparent inverse regulation by CjFur/CjPerR under iron limitation. RT-qPCR of selected genes in the Δ*fur* (Panel **a**), Δ*perR *(Panel **b**) and Δ*fur*Δ*perR *(Panel **c**) strains under iron limitation. Genes colored in blue show reduced expression in the Δ*fur* mutant, increased expression in the Δ*perR* mutant and no change in expression in the Δ*fur*Δ*perR* mutant in both the RNA-seq data and the RT-qPCR validation. Genes in green are differentially expressed in the Δ*perR*/Δ*fur*Δ*perR* mutants via RT-qPCR but only the Δ*perR* under RNA-seq. Cj1386 was found to be differentially expressed in the Δ*perR*/Δ*fur*Δ*perR* mutants under both techniques. *AhpC* and *chuA* are negative controls for metal dependant CjFur/CjPerR regulation. All expression values are relative to wild-type and normalized to *slyD*. Asterisks denote fold changes >1.5 with a *p* value <0.05

The results from the iron-independent RNA-seq experiments were confirmed using RT-qPCR (Fig. 4). Specifically, genes that appeared to show inverse CjFur/CjPerR regulation with no difference in the Δ*fur*Δ*perR* mutant were chosen for validation. As shown in Fig. 4, *flgE1*, *flgE2*, *flgH*, and *fliK* all showed the same expression pattern as identified using the RNA-seq analysis (*flgG* was the lone exception). Moreover *ahpC* and *chuA*, two well characterized metal dependent CjPerR and CjFur targets, were not differentially expressed in any of the strains under iron limitation asserting the experimental condition. In addition *cj0414*, *cj0415* and *cj1386* were confirmed to be differentially expressed in the Δ*perR* mutant. Interestingly, all three were also differentially expressed in the Δ*fur*Δ*perR* mutant while only the *cj1386* was identified using RNA-seq (although the *cj0414*/*cj0415* do show reduced expression in the Δ*fur*Δ*perR* mutant).

### Gene-set enrichment analysis of Δ*fur*, Δ*perR* and Δ*fur*Δ*perR* under both iron-rich and iron-limited conditions

**Fig. 5:**
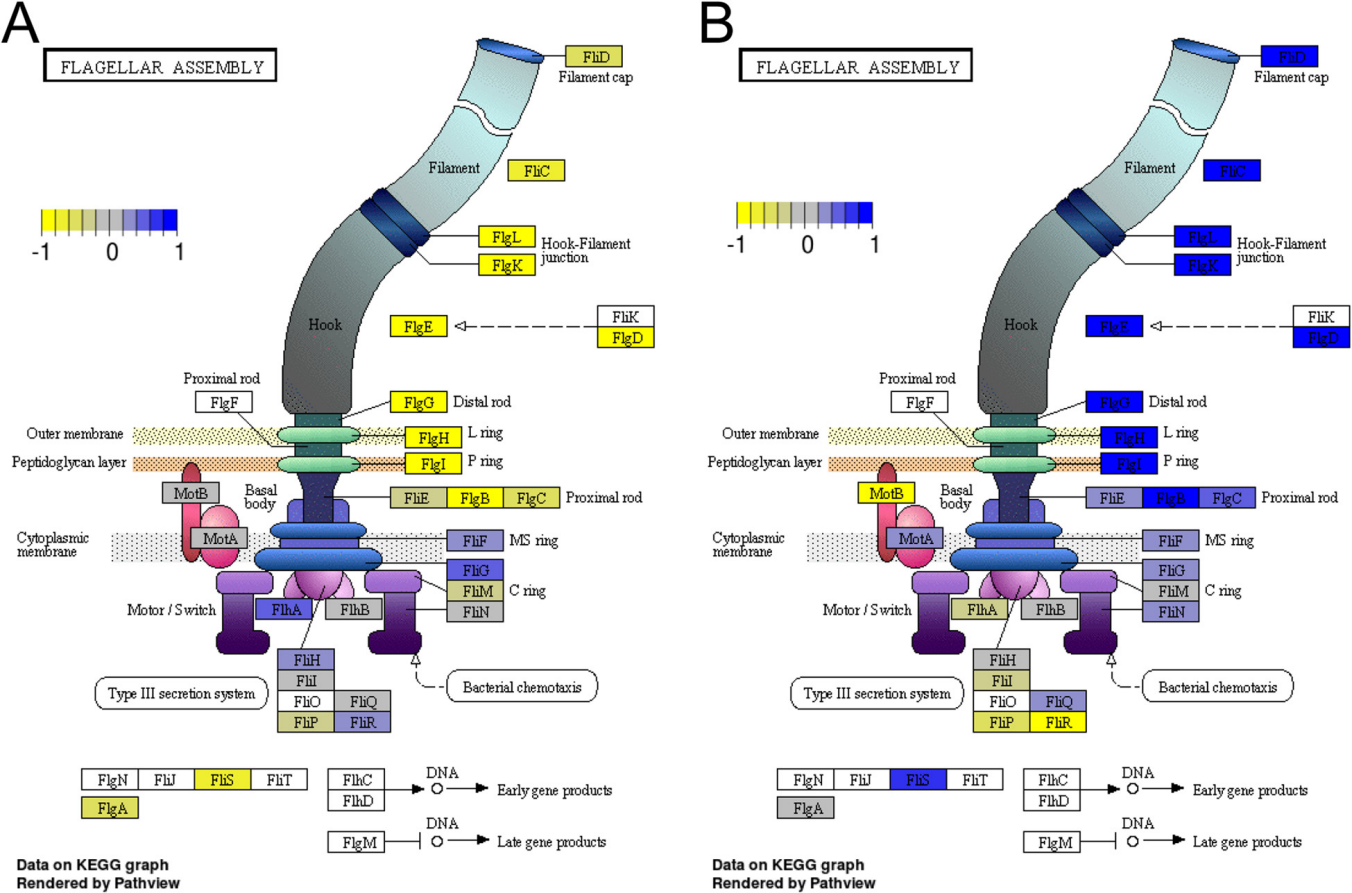
Genes enriched in KEGG’s “Flagellar Assembly” category under iron limitation. Genes that belong to the KEGG’s “Flagellar Assembly” category under iron-limited conditions in the Δ*fur* mutant (**a**) and Δ*perR* mutant (**b**) are shown. Genes absent from *C. jejuni* are denoted in white, unchanged genes in grey, up-regulated genes in blue and down-regulated genes in yellow. To note, the scale of differential expression is normalized from the highest to lowest expressed gene. Most of the genes down-regulated in the Δ*fur* mutant are conversely up-regulated in the Δ*perR* mutant

Gene-set enrichment analysis was used to determine if there were significantly enriched KEGG pathways present in the differentially expressed genes found in each strain under each growth condition. This analysis identified five KEGG categories that were enriched under at least one condition (Additional file 14: Table S8 and Additional file 15: Table S9). Interestingly, the KEGG category for “Flagellar Assembly” was enriched under both the Δ*fur* and Δ*perR* iron-limited conditions but not in the Δ*fur*Δ*perR* deletion mutant (Fig. 5).

### Cross platform correlation of the Δ*fur* and Δ*perR* transcriptomes

**Fig. 6:**
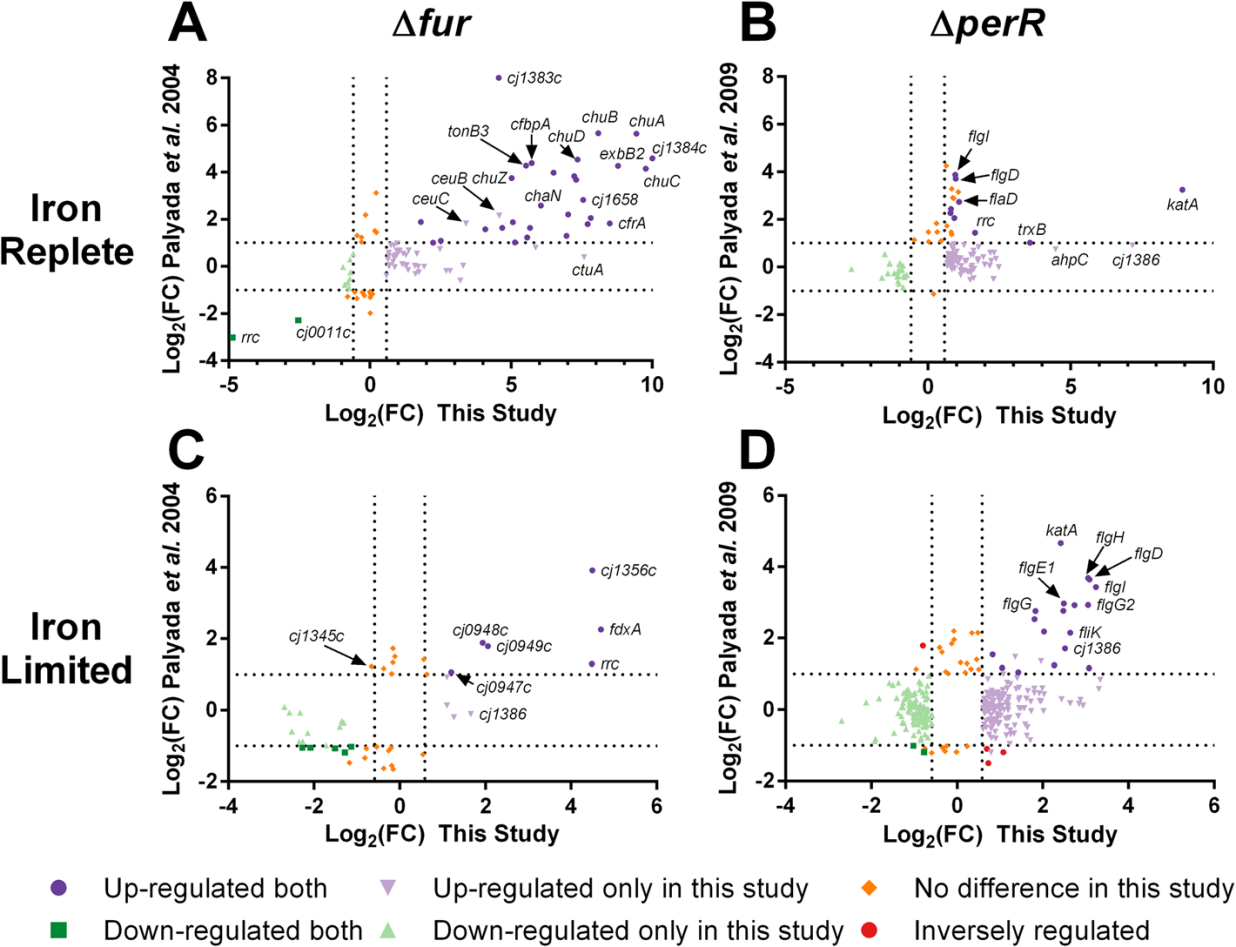
Comparison between CjFur and CjPerR transcriptomes as determined by RNA-seq and microarray profiling. The results of the RNA-seq in this study and previous CjFur (**a**, **c**) or CjPerR (**b**, **d**) microarray profiling results were plotted according to Log_2_(fold change). Only genes found to be differentially expressed in either the RNA-seq or microarrays are shown with the dashed lines representing the Log_2_(FC) cut-offs. Up-regulated genes are in purple and down-regulated genes in green. Genes not found to be differentially expressed in the present study are in orange and genes showing opposite regulation are in red. Genes mentioned in the text are highlighted. See Additional file 14: Table S8 for details for all differentially expressed genes

To validate the results from the RNA-seq analysis we compared the obtained CjFur and CjPerR transcriptomes to the transcriptomes previously defined using a microarray platform under similar growth conditions [2, 4]. The correlation between the two platforms was assessed by plotting the log-transformed values of the fold change obtained by RNA-seq against the log-transformed values of the fold change obtained by microarrays (only the differentially expressed genes identified by either platform were included). These two independent measures of differential gene expression showed poor correlation with correlation coefficients of 0.57/0.43 for Δ*fur* (iron-replete/limited) and 0.08/0.13 for Δ*perR* (iron-replete/limited). However, for both the Δ*fur* and Δ*perR* mutants there was a greater agreement between the genes identified as up-regulated as compared to down-regulated in each condition (Fig. 6, Additional file 14: Table S8). In addition, the RNA-seq analysis identified more genes as being differentially expressed as compared to the microarrays: 86/31 vs 49/30 for Δ*fur* (RNA-seq vs microarray, iron-replete/limited) and 111/272 vs 23/49 for Δ*perR* (RNA-seq vs microarray, iron-replete/limited). For the Δ*fur* mutant under iron-replete conditions, genes commonly identified as up-regulated include most of the known iron acquisition pathways (*ceuBCE*, *chuABCDZ*, *cfrA*, *cfbpABC*, *chaN*/*ctuA*, *cj1658*/*p19*, *cj1660*-*1665*, *exbB1*/*D1*, *exbB2*/*D2*, *feoB*, *tonB3*), as well as the highly iron responsive *cj1383c*-*cj1384c* genes located upstream from *katA*. The only genes found to be downregulated in the Δ*fur* mutant by both platforms were *rrc*-*cj0011c*. Genes identified in the RNA-seq but not the microarray profiling include the iron acquisition proteins *ctuA*/*cj0178* (transferrin/lactoferrin) and *ceuBC* (enterobactin). The commonly identified up-regulated genes for the Δ*fur* mutant under iron-limited conditions include *rrc*, *fdxA*, *cj1386* and *cj0948c*-*0949c*.

The commonly identified up-regulated Δ*perR* genes under iron-replete conditions include the antioxidant genes *katA*, *trxB* and *rrc* as well as a variety of genes involved in flagellar biogenesis (e.g. *flaD*, *flgD*, *fgI*). In contrast with the microarray profiling, the RNA-seq analysis also identified the antioxidant genes *ahpC* and *cj1386* as being CjPerR regulated. There were no CjPerR activated genes in common between the RNA-seq and microarray profiling under iron-replete conditions. The RNA-seq analysis for the Δ*perR* mutant under iron-limited conditions identified many differentially expressed genes that were not present in the microarray profiling. Many of the commonly identified up-regulated genes are also differentially expressed under iron-replete conditions (e.g. *katA*, *cj1386*, *flgI*, *flgD*). The Δ*perR* mutant under iron-limited conditions was also the only condition where there was a disagreement between the RNA-seq and microarray data for whether a gene was significantly up-regulated or down-regulated upon *perR* deletion (Fig. 6).

### Comparison of the iron regulon from two independent RNA-seq experiments and microarray profiling

**Fig. 7:**
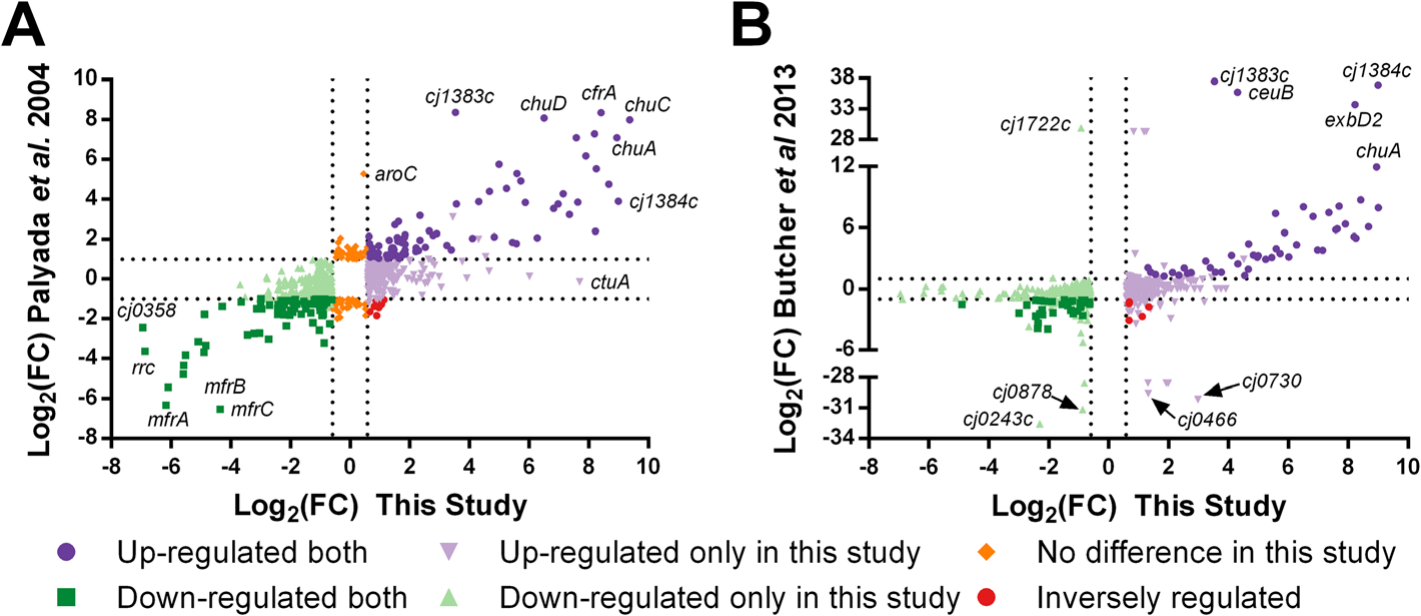
Comparison between iron transcriptomes determined in this work and previous studies. The results of the iron transcriptomes as determined in in this study and previous microarray (**a**) and RNA-seq (**b**) results were plotted according to Log_2_(fold change). Only genes found to be significantly expressed in each study are shown with the dashed lines representing the Log_2_(FC) cut-offs. Up-regulated genes are in purple with down regulated genes in green. Genes not found to be differentially expressed in the current study are in orange and genes showing inverse regulation are in red. See Additional file 10: Table S7 for details for all differentially expressed genes

Two previous studies from our laboratory have used similar growth conditions to determine iron-responsive genes in *C. jejuni* using both microarray profiling and RNA-seq [2, 7]. Iron-responsive genes were determined by comparing the wild-type transcriptomes in the presence and absence of iron. This analysis found 686 genes differentially expressed in response to iron as compared to 270 genes identified using microarray profiling and 127 genes identified in an earlier RNA-seq study (Fig. 7, Additional file 16: Table S10, Additional file 10: Table S7).

Overall, the RNA-seq analysis identified more differentially expressed genes as compared to the microarray analysis. Approximately 26 % of the genes identified in the RNA-seq analysis were also found to be differentially expressed in the microarray profiling experiments (180/686). In addition, there were 75 genes identified in the microarray analysis that were not found to be differentially expressed via RNA-seq (Additional file 10: Table S7).

In contrast, all of the genes identified as being iron regulated in a previous RNA-seq study were also found to be differentially expressed in this work [7]. However, the fold changes determined in this previous RNA-seq study appear to be greater as compared to this work. In particular, the Log_2_(FC)’s in the previous study exceeded >25 in some cases as compared to a maximum of Log_2_(FC) of ~10 in this work (Fig. 7). Indeed, most of these extremely differentially expressed genes (e.g. *cj0243c*, *cj0878*, *cj0466*, *cj0730*, *cj1722*) were not identified as significant in the previous RNA-seq study due to high variability in the number of reads across biological replicates [7]. However, all these genes were found to be significantly differentially expressed in this dataset.

## Discussion

Although the importance of the CjFur and CjPerR transcriptional regulators on *C. jejuni*’s *in vivo* fitness is now well established, there are still gaps in our understanding of their regulatory networks. Importantly, and in contrast to many other bacteria, CjFur regulates gene expression in an iron independent manner. However it is unknown whether CjPerR is also able to regulate genes independently from iron. As well, the degree to which the CjFur/CjPerR transcriptomes overlap is unclear as the transcriptome of a double deletion of *fur* and *perR* has not been described. In this study, we have used RNA-seq to profile the transcriptomes of Δ*fur*, Δ*perR* and Δ*fur*Δ*perR* isogenic deletion mutants in order to characterize the CjFur and CjPerR transcriptomes under both iron-replete and iron-limited conditions. In contrast to previously reported results, we have found that the transcriptomes for both of these metalloregulators are more extensive than anticipated. We have also found that these transcriptomes appear to reveal roles for both proteins in iron-dependant and iron-independent regulatory functions. Our work also highlights the importance of having multiple biological replicates when conducting genome wide analyses and to identify and remove outlier samples that may mask expression differences.

Unsurprisingly, the most up-regulated genes under iron-replete conditions in the isogenic deletion mutants are those previously characterized as being involved in iron acquisition (Fig. 2 [Cluster G]) or in oxidative stress defense (Fig. 2 [Cluster H]). These two functions were clearly separated with the iron acquisition genes up-regulated in the Δ*fur* mutant and the oxidative stress defense genes up-regulated in the Δ*perR* mutant. The Δ*fur* mutant had relatively few genes down-regulated under iron-replete conditions with only *rrc* and *cj0011c* forming a distinct CjFur activated cluster. In contrast, there were many more genes down-regulated in the Δ*perR* mutant and in the Δ*fur*Δ*perR* mutant (Fig. 2 [Clusters BC]), suggesting that PerR may also activate gene expression in an iron-dependent form.

Interestingly there were a large number of genes differentially expressed in the isogenic deletion mutants under iron-limited conditions, which was unexpected as both CjFur and CjPerR are metalloregulators. In particular, there were 31 and 272 genes differentially expressed in the Δ*fur* and Δ*perR* mutants respectively (Fig. 3). While CjFur has been previously shown to regulate gene expression in an iron-independent manner, this result suggests that CjPerR may also regulate genes independently from iron. However, additional studies using purified CjPerR protein without a metal cofactor would be required to demonstrate this specific mode of regulation as transcriptomic studies cannot distinguish between direct and indirect regulation.

Under iron-limited conditions the Δ*fur* mutant also showed relatively few down-regulated genes as compared to up-regulated genes. The oxidative stress defense genes *cj1386*, *fdxA* and *rrc* were found to be regulated by CjFur independently from iron. *Rrc* has been previously shown to be differentially expressed in a Δ*fur* mutant under iron-limited conditions by RT-qPCR and directly bound by metal free CjFur [6]. Previous work has also shown *cj1345c* to be differentially expressed in Δ*fur* mutant under iron-limited conditions using RT-qPCR and also shown to be directly bound by metal free CjFur via EMSA [6]. However in this study, *cj1345c*’s change in expression level was not statistically significant. There was also a cluster of genes that appear to be up-regulated in the Δ*fur* mutant and down-regulated in the Δ*perR* mutant under iron-limited conditions (Fig. 3 [Cluster E], Additional file 12: Figure S6). Interestingly these genes are primarily composed of flagellar biogenesis genes (*flgD*, *flgE1*, *flgE2*, *flgG2*, *flgH*, *flgI*, *flgJ*, *fliK*). Indeed, GSEA found that the KEGG category for flagellar assembly was enriched in both the Δ*fur* and Δ*perR* iron-limited conditions, with most of the flagellar assembly apparatus under opposing regulation in these two mutants (Additional file 14: Table S8, Additional file 15: Table S9, Fig. 5). Further supporting the fact that these genes appear to be differentially regulated by CjFur/CjPerR is the fact that these genes were not differentially expressed in the Δ*fur*Δ*perR* mutant (Additional file 12: Figure S6), suggesting that each protein exerts its influence independently and GSEA did not identify flagellar assembly as being significantly enriched in the Δ*fur*Δ*perR* mutant (Additional file 14: Table S8, Additional file 15: Table S9). This represents another manner by which the CjFur and CjPerR transcriptomes may overlap with each other. In the other cases where CjFur and CjPerR are known to co-regulate the same gene (e.g. *katA* repression), both transcriptional regulators have the same effect on transcription. These interactions between the CjFur and CjPerR transcriptomes further contrast with other bacteria, where their corresponding Fur and PerR transcriptomes are separate from each other [11]. It should also be noted that *C. jejuni perR* has also been shown to be autoregulated and thus this may account for some of the differences seen in the Δ*perR* mutant [4, 29].

When comparing the transcriptomes identified in this study using RNA-seq (CjFur, CjPerR and iron) to those identified using microarray profiling the most obvious observation is that the RNA-seq identified substantially more differentially expressed genes as compared to the microarray profiling (Fig. 6). This is likely due to several factors. Firstly, the greater dynamic range offered by RNA-seq allows for the assignment of genes being differentially expressed at a lower fold change as compared to microarrays (1.5 fold as compared to 2 fold) which naturally leads to more genes being assigned as differentially expressed. Indeed, most of the genes identified using both techniques were those that showed the greatest level of differential expression. In addition, microarrays often have difficulties identifying differentially expressed genes which are expressed at high levels in both the test and control conditions due to signal saturation. For example, previous microarray profiling did not detect any significant change in *ceuBC*/*ctuA* in the Δ*fur* mutant and *ahpC* in the Δ*perR* mutant [2, 4]. This is despite the presence of Fur/PerR boxes in each promoter region and gel shift assays showing that each promoter was directly bound by either CjFur/CjPerR [8, 27, 29]. As RNA-seq works by directly counting the reads aligning to each gene it does not suffer from this same limitation and correctly identified these genes as being differentially expressed in the CjFur/CjPerR mutants respectively.

In this study, PCA analysis was used to determine the relative similarity of each strain’s biological replicates. This analysis identified two samples that appeared to be substantially different from their mates and that were subsequently discarded in the downstream analyses. Including these samples in the differential expression analysis leads to markedly different results depending on each strain. Including the erroneous Δ*perR* iron-replete sample in the differential analysis leads to an iron-limited gene profile (i.e. all iron acquisition genes become significantly up-regulated) whereas including the erroneous Δ*fur* iron-limited sample leads to essentially no genes being identified as differentially expressed (data not shown). The potential for variability between samples influencing results is highlighted when comparing the iron regulon determined in this study with a previously published dataset [7]. In the previous study, the iron-replete samples showed a greater level of variability (Additional file 11: Figure S4) that resulted in many genes not being identified as significantly differentially expressed. However, it should be noted that all the genes actually identified as being iron regulated were confirmed in this work. Moreover, while some of the variability present between these aforementioned samples could be deduced by plotting sample RPKMs and determining the R^2^ value for each pair of replicates, some samples identified as outliers in the PCA analysis could not be confidently distinguished from their mates based on R^2^ values alone (data not shown). As such, higher order analyses for determining sample similarity (PCA vs R^2^ values etc.) and multiple (>2) replicates for different samples are highly recommended for future studies employing RNA-seq for differential gene expression analysis. Another factor that could influence the effectiveness of RNA-seq analyses is the number of reads aligning to coding regions (i.e. sequencing depth). While sequencing depth is a difficult concept to define in transcriptomic studies (due to variations in the complexity of each unique transcriptome), increasing the number of reads aligning to each gene will likely increase the confidence in the downstream analyses [32]. The number of reads aligning to genes is 10x greater in this current study as compared to the previous RNA-seq study identifying iron regulated genes in *C. jejuni* [7]. This greater sequencing depth appears to have more successfully captured the distribution of transcripts expressed at low levels in either iron-replete/iron-limited conditions and resulted in attenuated fold changes as compared to previous work (Fig. 7, Panel b).

## Conclusions

In conclusion, our results indicate that both the CjFur and CjPerR transcriptomes are larger than previously believed. In particular, deletion of *perR* appears to result in the differential expression of a large group of genes in the absence of iron, suggesting that CjPerR may also regulate genes in an iron-independent manner; similar to what has already been demonstrated with CjFur. We have also found subsets of genes which are only differentially expressed when both CjFur and CjPerR are deleted. Finally, we have genes which appear to be simultaneously activated by CjFur and repressed by CjPerR, which represents another manner in which these two transcriptomes interact with each other.

## Availability of supporting data

The raw sequencing reads for this study have been submitted to the Sequence Read Archive at NCBI (http://www.ncbi.nlm.nih.gov/sra) with the accession number SRP044881. Other supporting information is available in the supplemental files associated with this manuscript.

## Additional files

Additional file 1: Table S1. Statistics for high throughput sequencing runs. Additional file 2: Table S2. Total reads aligning to *Campylobater jejuni* NCTC11168 genes. Additional file 3: Table S3. RPKMs for *Campylobater jejuni* genes. Additional file 4: Figure S1. PCA analysis of *C. jejuni* transcriptomic samples by growth condition. The Log(RPKM + 1) values for each gene in the *C. jejuni* NCTC11168 genome were used for PCA analysis under iron-replete (AC) or iron-limited (BD) conditions to show the overall structure of the transcriptomic data and to identify sample outliers (highlighted by arrow). Under iron-replete conditions different strains cluster together with the exception of one of the Δ*perR* samples (this sample clusters with iron-limited samples in the expanded PCA analysis). The samples under iron-limited conditions do not cluster as tightly as the iron-replete conditions indicating greater variability in their transcriptomes. In addition, there appears to be an outlier within the iron limited Δ*fur* samples which clusters away from its mates in the PC1vsPC2 components (Panel B). Additional file 5: Table S4. Differentially expressed genes between the wild-type and various mutant strains. Additional file 6: Table S5. Differentially expressed genes under iron-replete conditions. Additional file 7: Table S6. Differentially expressed genes under iron-limited conditions. Additional file 8: Figure S2. Expanded hierarchical clustering of genes differentially expressed under iron-replete conditions. Genes found to be differentially expressed in at least one strain under iron-replete conditions were subjected to hierarchical clustering in Genesis to identify corresponding genes with similar expression profiles. The columns each represent one strain (Δ*fur*, Δ*perR*, Δ*fur*Δ*perR*) and relative fold changes in expression are presented in a Log_2_ scale with up-regulated genes in blue and down-regulated genes in yellow. The clustering resulted in 8 main clusters (A-H). See Additional file 6: Table S5 for further details. Additional file 9: Figure S3. Hierarchical clustering of genes differentially expressed under iron-limited conditions. Genes found to be differentially expressed in at least one strain under iron-limited conditions were subjected to hierarchical clustering in Genesis to identify corresponding genes with similar expression profiles. The columns each represent one strain (Δ*fur*, Δ*perR*, Δ*fur*Δ*perR*) and relative fold changes in expression are presented in a Log2 scale with up-regulated genes in blue and down-regulated genes in yellow. The clustering resulted in 11 main clusters (A-K). See Additional file 7: Table S6 for further details. Additional file 10: Table S7. Comparison between iron regulon in this study as compared to previous publications. Additional file 11: Figure S4. PCA analysis of previous RNA-seq samples. The Log(RPKM + 1) values for each gene in the *C. jejuni* NCTC11168 genome were used for PCA analysis from the wild-type samples in this study and Butcher et al. 2013 (AC) or in Butcher et al. alone (BD). When comparing both studies samples can be differentiated based on two main axes that correspond to growth condition (iron-replete vs iron limited) and study (current study vs previous study). With the exception of the iron-replete samples from Butcher et al. 2013, all the samples cluster closely with their biological replicates (outlier highlighted by arrow). When the wild-type samples from Butcher et al. are analyzed separately (BD) they separate based on growth condition, with no obvious biases between biological replicates. Additional file 12: Figure S5. Overlap between the genes differentially expressed in the Δ*fur*, Δ*perR* and Δ*fur*Δ*perR* strains. Hive cluster showing the relationship between genes differentially expressed in each strain under either iron-replete (A) or iron-limited (B) conditions. Each axis contains the genes found to be differentially expressed for the specific strain listed which are colored based on their mode of regulation (green for activation, purple for repression). Genes which are differentially expressed in multiple strains are connected by ribbons to highlight the similarities between the transcriptomes observed in each strain. Additional file 13: Figure S6. Iron-dependent CjFur repression binding motif. The iron-dependent CjFur repression motif contains an inverted palindrome (marked by arrows and centered at the asterisk) and is quite similar to those previously reported for iron-dependent CjFur repression [2, 6]. Additional file 14: Table S8. KEGG gene set enrichment analysis of upregulated genes. Additional file 15: Table S9. KEGG gene set enrichment analysis of downregulated genes. Additional file 16: Table S10. Comparison between RNA-seq Δfur/ΔperR differentially expressed genes and previous microarray results.
