# Supplementary figures and images for: Refined analysis of the Campylobacter jejuni iron-dependent/independent Fur- and PerR-transcriptomes
Source: BMC Genomics. 2015 Jul 4;16(1):498. doi: 10.1186/s12864-015-1661-7 (PMC4491227; doi:10.1186/s12864-015-1661-7)

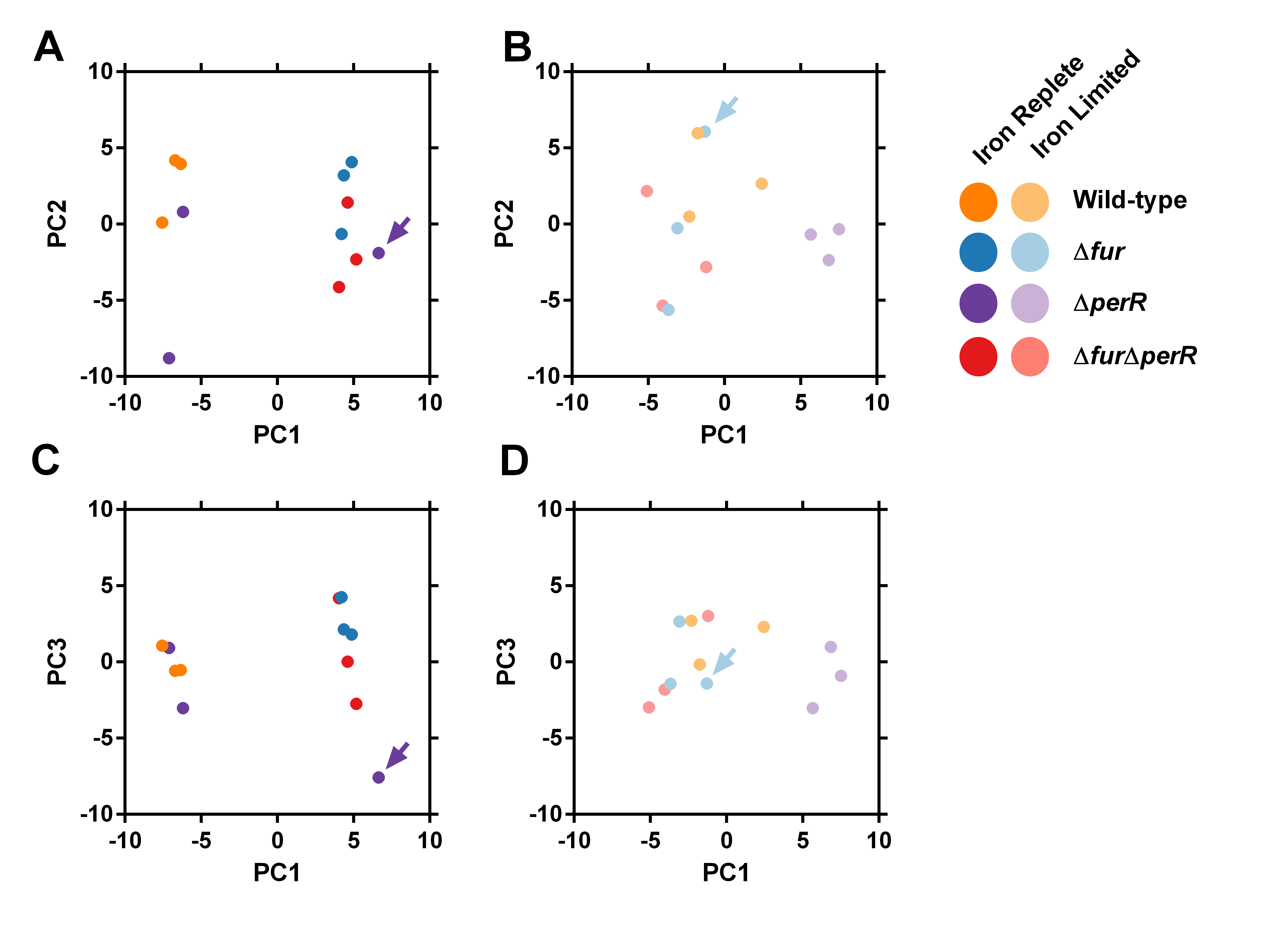

Supplement: Additional file 4: Figure S1. — PCA analysis of C. jejuni transcriptomic samples by growth condition. The Log(RPKM + 1) values for each gene in the C. jejuni NCTC11168 genome were used for PCA analysis under iron-replete (AC) or iron-limited (BD) conditions to show the overall structure of the transcriptomic data and to identify sample outliers (highlighted by arrow). Under iron-replete conditions different strains cluster together with the exception of one of the ΔperR samples (this sample clusters with iron-limited samples in the expanded PCA analysis). The samples under iron-limited conditions do not cluster as tightly as the iron-replete conditions indicating greater variability in their transcriptomes. In addition, there appears to be an outlier within the iron limited Δfur samples which clusters away from its mates in the PC1vsPC2 components (Panel B). [file 12864_2015_1661_MOESM4_ESM.tiff]

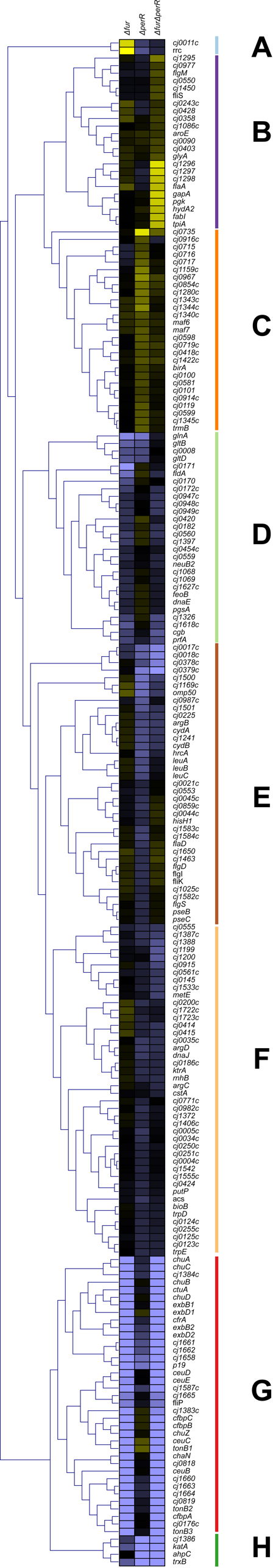

Supplement: Additional file 8: Figure S2. — Expanded hierarchical clustering of genes differentially expressed under iron-replete conditions. Genes found to be differentially expressed in at least one strain under iron-replete conditions were subjected to hierarchical clustering in Genesis to identify corresponding genes with similar expression profiles. The columns each represent one strain (Δfur, ΔperR, ΔfurΔperR) and relative fold changes in expression are presented in a Log2 scale with up-regulated genes in blue and down-regulated genes in yellow. The clustering resulted in 8 main clusters (A-H). See Additional file 6: Table S5 for further details. [file 12864_2015_1661_MOESM8_ESM.tiff]

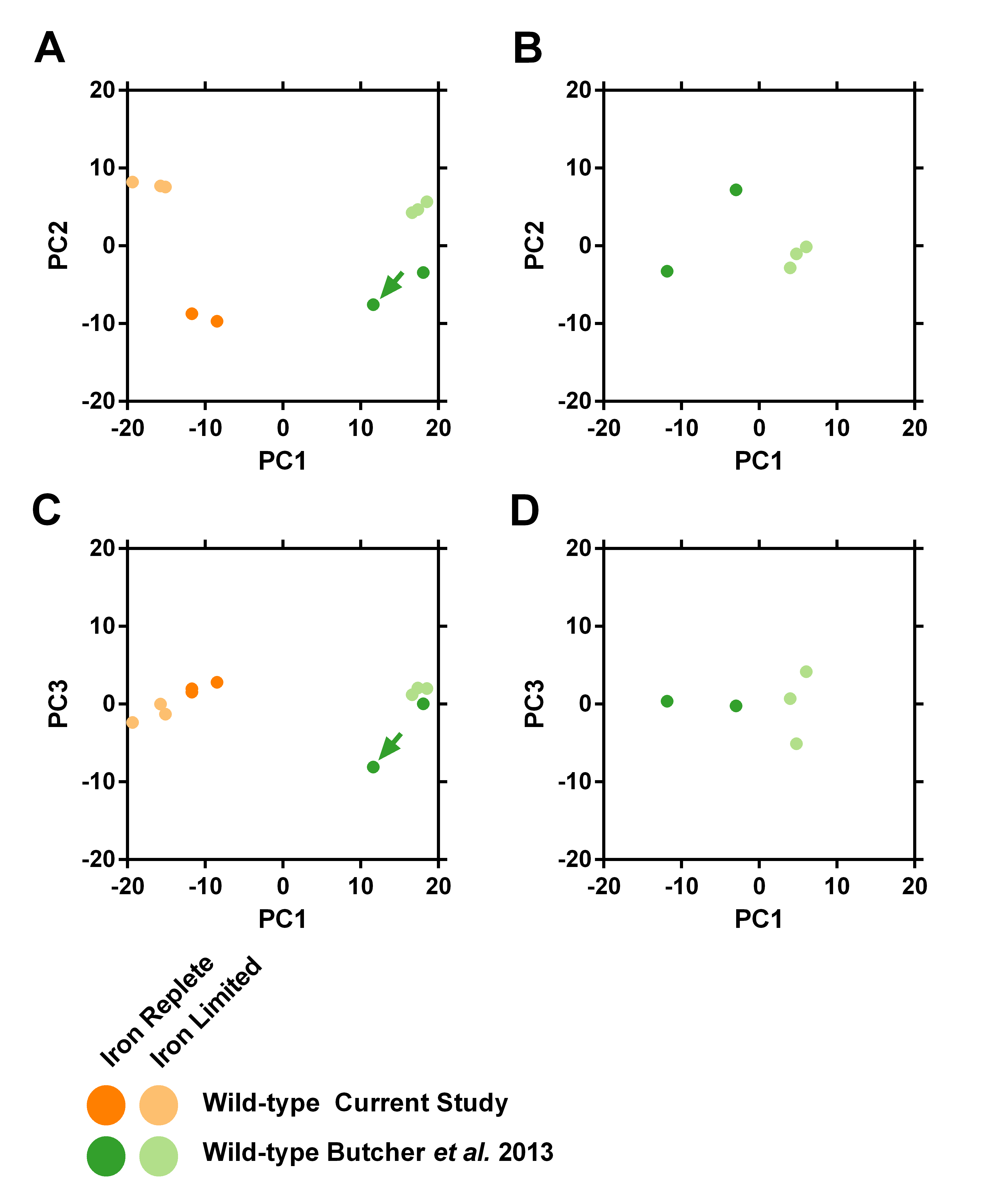

Supplement: Additional file 11: Figure S4. — PCA analysis of previous RNA-seq samples. The Log(RPKM + 1) values for each gene in the C. jejuni NCTC11168 genome were used for PCA analysis from the wild-type samples in this study and Butcher et al. 2013 (AC) or in Butcher et al. alone (BD). When comparing both studies samples can be differentiated based on two main axes that correspond to growth condition (iron-replete vs iron limited) and study (current study vs previous study). With the exception of the iron-replete samples from Butcher et al. 2013, all the samples cluster closely with their biological replicates (outlier highlighted by arrow). When the wild-type samples from Butcher et al. are analyzed separately (BD) they separate based on growth condition, with no obvious biases between biological replicates. [file 12864_2015_1661_MOESM11_ESM.tiff]

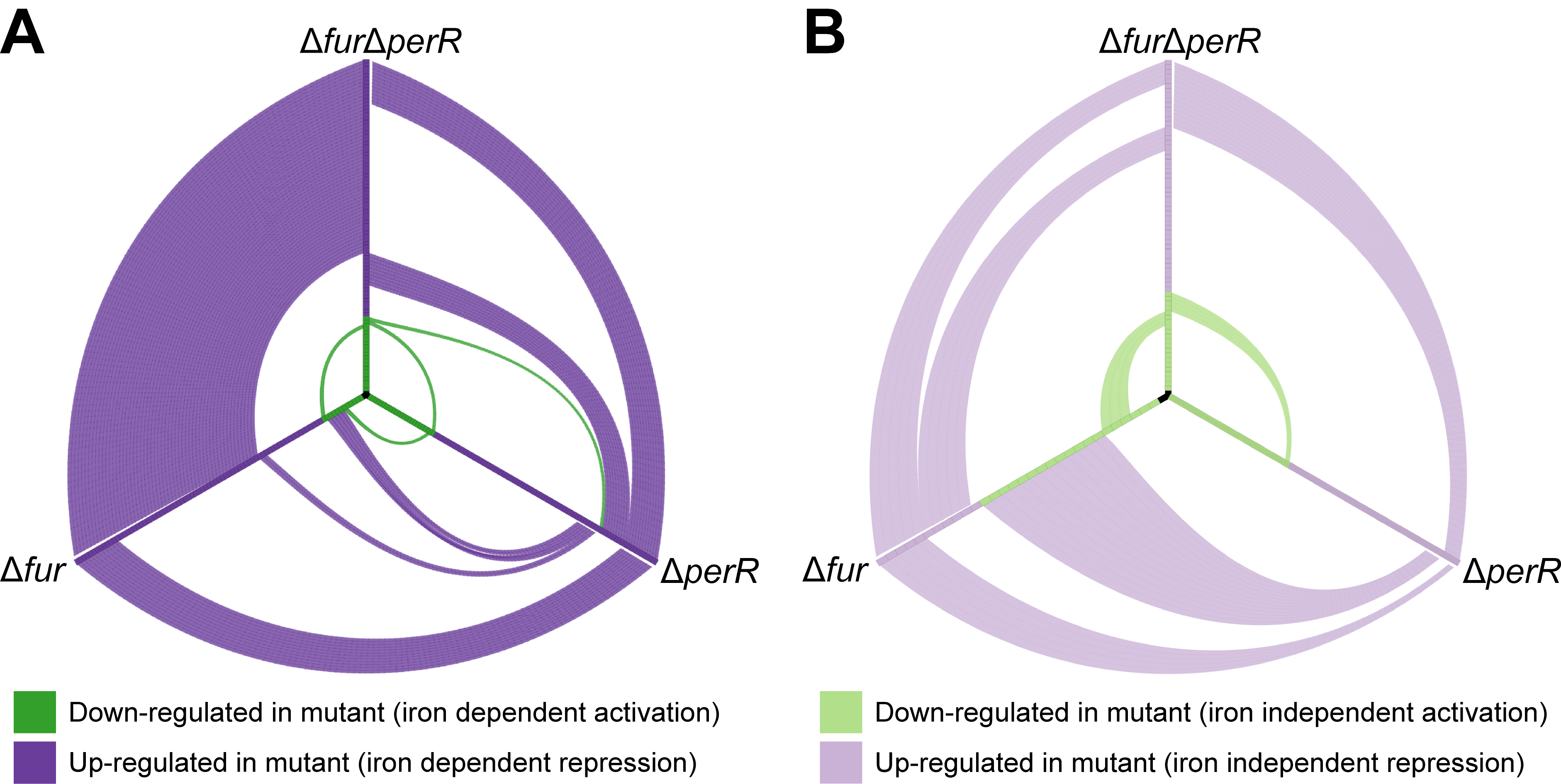

Supplement: Additional file 12: Figure S5. — Overlap between the genes differentially expressed in the Δfur, ΔperR and ΔfurΔperR strains. Hive cluster showing the relationship between genes differentially expressed in each strain under either iron-replete (A) or iron-limited (B) conditions. Each axis contains the genes found to be differentially expressed for the specific strain listed which are colored based on their mode of regulation (green for activation, purple for repression). Genes which are differentially expressed in multiple strains are connected by ribbons to highlight the similarities between the transcriptomes observed in each strain. [file 12864_2015_1661_MOESM12_ESM.tiff]

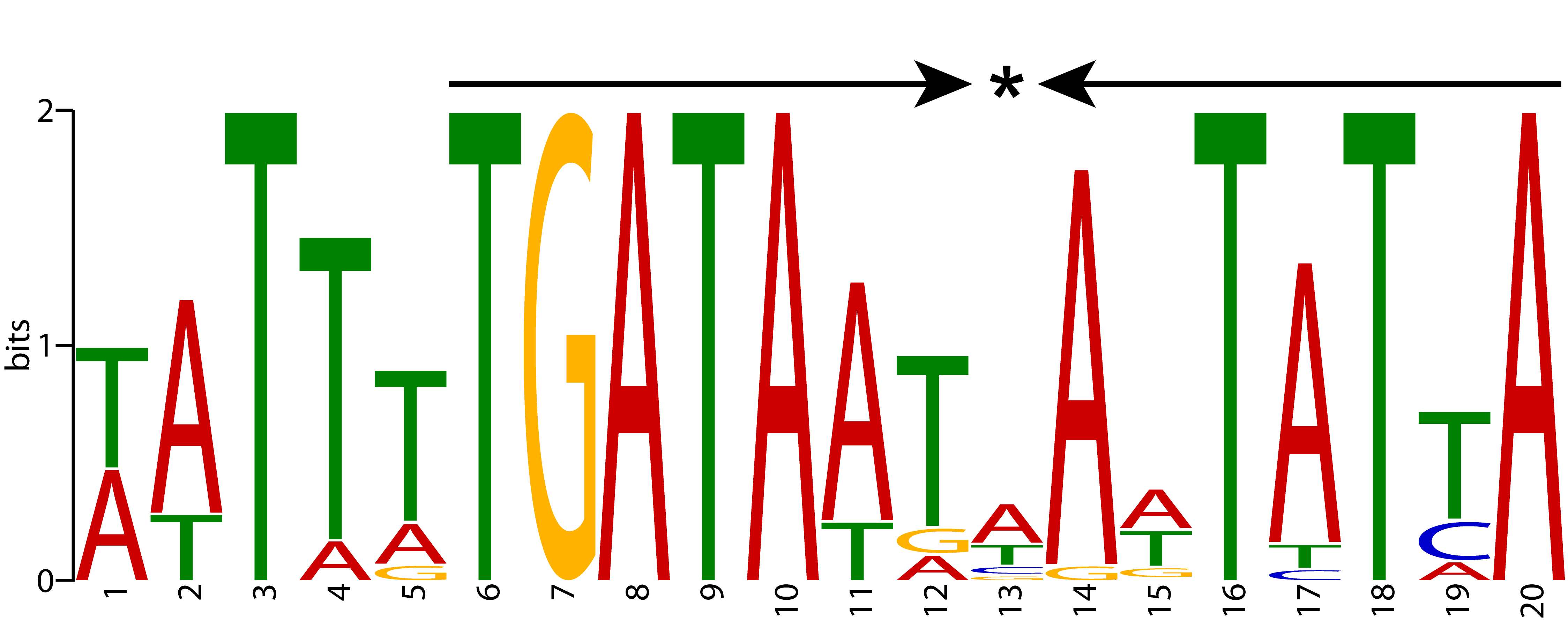

Supplement: Additional file 13: Figure S6. — Iron-dependent CjFur repression binding motif. The iron-dependent CjFur repression motif contains an inverted palindrome (marked by arrows and centered at the asterisk) and is quite similar to those previously reported for iron-dependent CjFur repression [2, 6]. [file 12864_2015_1661_MOESM13_ESM.tiff]
